# Atypical presentation of hemorrhagic shock in pregnancy: a case highlighting the developing field of emergency medicine in Israel

**DOI:** 10.1186/s12873-019-0272-z

**Published:** 2019-11-21

**Authors:** Barcuh Berzon, Michael Gleenberg, Joseph Offenbacher, Debra West

**Affiliations:** 1 Department of Emergency Medicine Ha-Refu’a St 7, Samson Assuta Ashdod Medical Center, 7747629 Ashdod, Israel; 2 Albert Einstein College of Medicine, Department of Emergency Medicine at the Jacobi and Montefiore Hospitals 1400ham Pkwy S, Bronx, NY 10461 USA

**Keywords:** Shock in early pregnancy, Early uterine rupture, International emergency medicine, Point of care ultrasonography, Resuscitation

## Abstract

**Background:**

Occult hemorrhagic shock secondary to uterine rupture represents a true obstetric emergency and can result in significant morbidity and mortality for both the patient and the fetus. Multiparity and prior cesarean sections are known risk factors. Typically, these patients present late in gestation, often secondary to the physiologic stresses on the uterus related to contractions. This pathology is less common earlier in pregnancy and can often be overlooked in the acute setting.

**Case presentation:**

We present the case of a 31-year-old female with three prior gestations, two parities and two prior cesarean sections, resulting in three live births, who presented to the Emergency Department (ED) 22-weeks pregnant with acute onset dyspnea and an episode of syncope. Due to her altered mental status there was concern for occult shock, despite normal vital signs. Large amounts of free fluid in the abdomen were noted on bedside ultrasonography with a high suspicion for uterine pathology. She was resuscitated with blood and taken immediately to the operating room for surgical management where she was found to have had a uterine rupture.

**Conclusion:**

This case highlights a rare presentation of a well-known obstetric emergency, due to the patient’s development of uterine rupture early in gestation. Consequently, emergency physicians should consider atraumatic hypovolemic shock, secondary to this obstetric catastrophe, even at a stage that far precedes its expected presentation. In addition, we make note of how this case validated our department’s integrated emergency medicine model, the first in the State of Israel.

## Background

Uterine rupture represents a true obstetric emergency and is known to result in significant morbidity and mortality secondary to hemorrhagic shock for both the patient and the fetus [1]. Multiparity and prior cesarean sections are known risk factors. Prompt identification of this underlying disease process is critical. Furthermore, mobilizing interdisciplinary teams of resuscitationists and obstetricians to manage the cardiovascular collapse and to surgically repair the underling pathologic process is essential.

Typically, the diagnosis of uterine rupture is made in women presenting late in gestation with vaginal bleeding and abdominal pain. Often it results secondary to the physiologic stresses on the uterus related to peripartum contractions during labor and is rarely seen, even in labor, in patients earlier than 28 weeks gestation. Due to rarity, it is a diagnosis that is often missed in the acute setting.

## Case presentation

A 31-year-old, gravada-three, para-two, female with two prior cesarean sections and three live births, at 22-weeks gestation presented to the Emergency Department (ED) via ambulance with a chief complaint of severe dyspnea. The patient had no other past medical history and no prior obstetric complications. She was reported to have had one episode of syncope lasting several seconds, with no head trauma. At the time of presentation, the patient did not have, or report any prior, abdominal pain. However, when obtaining additional history from the patient’s husband, it was reported that the patient had experienced a brief episode of abdominal pain prior to her syncope. There were no other pertinent findings on history of present illness or review of symptoms.

On physical exam, the patient appeared pale and her skin was cool to touch. Her pulse was 72 beats/minute and regular, blood pressure was 110/80 mmHg, oxygen saturation was 97% on room air and her oral temperature was 37 C. She had altered mental status with intermittent episodes of drowsiness and restlessness. The remainder of her exam was significant for a gravid uterus slightly above the umbilicus with no significant tenderness elicited on palpation. Vaginal exam was negative for blood in the vault. Despite the patient’s normal blood pressure, her altered mental status combined with her cold and clammy extremities were consistent with compensated shock commonly manifesting with cutaneous and neurologic findings preceding hemodynamic instability.

As part of the patient’s initial management, laboratory studies were drawn. Significant findings included a white blood cell count of 24.4 × 10^3^/mm^3^. Her initial hemoglobin of 9.8 mg/dL later fell to 7.9 mg/dL. A venous blood gas included a pH of 7.295 and a lactate of 4 mmol/L. Of note, however, was that the majority of these laboratory studies had not yet resulted prior to the patient’s initial management and disposition, and were generally noncontributory to her care in the emergency department.

Due to the patient’s clinical presentation of undifferentiated shock, a point of care ultrasound (POCUS) was immediately done and revealed a significant amount of fluid in the abdominal cavity along with fetal bradycardia. Secondary to these findings, an intra-abdominal bleed was suspected and fluid resuscitation with unmatched uncrossed blood (O-negative) was initiated.

The obstetric service (OB/GYN) was urgently consulted and a repeat bedside ultrasound, performed by the OB/GYN attending, concurred with our findings of intraabdominal hemorrhage but was not diagnostic for uterine pathology or a clear source of the bleeding. It did, however, show that there was no fetal heart beat or movement, concerning for fetal demise. The patient was taken for emergent surgical exploration that showed an acute left uterine wall rupture extending into the parametrium with an invasion of the placenta. Three liters of blood were evacuated from the abdominal cavity, as well as the pre-viable pregnancy. After separating the placenta from the uterus, the uterus was surgically repaired and a surgical drain was left in place. The patient required eight units of packed red blood cells and four units of fresh frozen plasma over the course of her resuscitation. She ultimately had an uneventful medical recovery with discharge on post-operative day five.

## Discussion and conclusions

Spontaneous uterine rupture is rare, with a reported incidence of 0.5% of all pregnancies, and catastrophic complication, with significant morbidity and mortality for both the mother and fetus [2]. The patient’s previous deliveries by cesarean section were her only major risk factors for spontaneous uterine rupture in subsequent pregnancies [3]. There are, however, reports of uterine rupture in the unscarred uterus and even in nulliparous women [4, 5]. Furthermore, the most common clinical presentation of spontaneous uterine rupture, as described in the literature, is during active labor. There are, however, limited reports of this process occurring at earlier stages of pregnancy [6]. When uterine rupture does present during early trimesters it is most often seen in women with a scarred uterus secondary to prior surgeries involving the uterus or with underlying physiologic defects such as placenta precreta. In these instances, patients can have defects of the myometrium and endometrium compromising the uterus’ anatomic structural integrity [7].

The low incidence of uterine rupture prior to the third trimester, when considered in the context of more common etiologies of shock in pregnancy such as acute pulmonary embolism, often pushes this diagnosis further down the differential, even in patients whose shock state is more clinically consistent with an underlying hypovolemic etiology. However, even though uterine rupture is rare prior to the third trimester, it should be considered in the differential diagnosis of a gravid patient as early as the second trimester [8]. Failure in diagnoses can lead to catastrophic morbidity and even mortality for both the mother and the fetus.

Current literature pertaining to early uterine rupture prior to the third trimester in the emergency medicine setting is limited to a small number of case reports [9, 10]. Together these reports highlight how easily hemorrhagic shock can be overlooked, in this patient population, with devastating consequences. In one such report a patient with a strong clinical picture for hemorrhagic shock was sent for a pulmonary embolism imaging study, due to the latter diagnosis being more common. This patient subsequently developed cardiac arrest secondary to the delayed resuscitation [11].

Many reports demonstrate poor maternal outcome often secondary to misdiagnosis as a consequence of pursuing other, seemingly more common, underlying etiologies. One report highlighted a series of cases with patients presenting between 14 and 24 weeks gestation. In each instance infectious, or other non-gynecologic pathologies were mistakenly diagnosed rather than uterine rupture. In one case, a patient who was 14 weeks pregnant, with acute abdominal pain following intercourse, a primary gynecologic pathology was dismissed after an intrauterine pregnancy was confirmed by bedside ultrasonography. Only later, on autopsy, was the uterine rupture revealed. In a second case, suspected acute gastroenteritis with hemodynamic compromise was later discovered, on laparotomy, to have in fact been uterine rupture. Outcomes from these cases strongly suggested the need to consider, and rule out, this pathology regardless of gestational age [12].

An additional report by *Sung etal* described a case of a patient, fourteen weeks pregnant, who presented with acute abdominal pain. As with prior cases, the early time course and the reassurance of an intrauterine pregnancy on ultrasonography steered the team away from the diagnosis of uterine rupture. The patient was taken for laparoscopy to evaluate for, “appendicitis, cholecystitis, and peritonitis” when the uterine pathology was incidentally discovered [13]. Additional case reports further highlight the risk of this pathology in the context of underlying placenta precreta and similarly advocate for a high level of suspicion for uterine rupture even at earlier stages of pregnancy [14].

In addition to its clinical ramifications, we also highlight this case due to its unique role in the development of our department on an institutional and national level. Emergency Medicine is a young specialty in Israel and indeed in many countries throughout the world. Despite, and perhaps because of, our country’s already strong and well-established healthcare system, we have been slow to adopt the fully integrated emergency medicine model into our broader house of medicine and national healthcare design. While our specialty is fully recognized by the Ministry of Health, and we are rapidly developing in clinical practice, undergraduate and graduate medical education, and research, our hospital is currently the only one in the nation with 24-hour, seven-day-a-week, coverage by board-certified emergency medicine physicians.

At its foundation, the case presented above is one of a patient with undifferentiated compensated circulatory shock. The shock state itself was extremely subtle requiring specialists in resuscitation, such as our board-certified emergency medicine specialists, to identify. Furthermore, occult shock in pregnancy can represent a broad differential diagnosis and a underlying pathologic state, such as a pulmonary embolism, that may not warrant OBGYN management. Therefore, diagnostic generalists, such as emergency physicians, represent an appropriate first line in evaluating and properly diagnosing the underlying disease process. Finally, once a diagnosis is made early resuscitation is often needed to enable interventional specialists, such as OBGYN physicians, to proceed with definitive management.

In light of the above, we believe that the integrated EM model of care practiced in our hospital played a crucial role in the prompt diagnosis and management of this patient. The unique presentation of this patient and the need for both expert resuscitation and coordinated definitive management exemplified the need for emergency medicine specialists operating in a fully integrated emergency department. Our patient’s total “Door to Knife” time was 25 minutes. In that time, she was seen by a board-certified emergency physician, correctly identified despite her very subtle presentation of acute shock via both clinical expertise and bedside imaging, resuscitated and dispositioned to a coordinated multidisciplinary team for definitive management.

This case provides an important insight into both the diagnosis and management of a unique pathologic presentation, as well as underscoring the need for systems-based approaches to emergency care. Clinically, it highlights the need to maintain a broad differential diagnosis for the pregnant patient who presents acutely ill even at earlier stages in pregnancy. While obstructive shock secondary to pulmonary embolism must be highly considered, even in the second trimester, hypovolemic shock with intra-abdominal bleeding secondary to a uterine catastrophe must not be overlooked. Additionally, this case, and others like it, specifically highlight the unique role our specialty plays in the appropriate diagnosis, management and integrated disposition of acutely ill patients. As the field of emergency medicine continues to develop, throughout the world, these types of cases provide both external validation and internal confirmation of our practice model and should continue to be highlighted.

Ultimately, circulatory compromise in a pregnant patient early in gestation presents a unique diagnostic and management challenge for physicians in the emergency department due to their increased incidence of obstructive, cardiogenic and even hemorrhagic shock. Uterine rupture is an obstetric catastrophe with extremely high morbidity and mortality secondary to hemorrhagic shock that can be easily overlooked early in pregnancy. We feel that this case highlights the need for emergency physicians to consider this atypical diagnostic presentation due to its potential for morbidity and mortality, while providing further insight into the meaningful impact our specialty’s approach has in the context of developing emergency medicine systems.

## Data Availability

Not applicable in our case report.

## Abbreviations

ED: Emergency Department
OBGYN: Obstetrics and Gynecology
POCUS: Point of Care Ultrasound
