# Correction to: Atypical presentation of hemorrhagic shock in pregnancy: a case highlighting the developing field of emergency medicine in Israel

**DOI:** 10.1186/s12873-020-0301-y

**Published:** 2020-01-15

**Authors:** Baruch Berzon, Michael Gleenberg, Joseph Offenbacher, Debra West

**Affiliations:** 1 Department of Emergency Medicine Ha-Refu’a St 7, Samson Assuta Ashdod Medical Center, 7747629 Ashdod, Israel; 2 Albert Einstein College of Medicine, Department of Emergency Medicine at the Jacobi and Montefiore Hospitals 1400ham Pkwy S, Bronx, NY 10461 USA

**Correction to: BMC Emerg Med (2019) 19:70**

10.1186/s12873-019-0272-z

The original article [1] contained a misspelling in first author, Baruch Berzon’s name which has since been corrected.
